# Cognitive rejuvenation in old rats by hippocampal OSKM gene therapy

**DOI:** 10.1007/s11357-024-01269-y

**Published:** 2024-07-22

**Authors:** Steve Horvath, Ezequiel Lacunza, Martina Canatelli Mallat, Enrique L. Portiansky, Maria D. Gallardo, Robert T. Brooke, Priscila Chiavellini, Diana C. Pasquini, Mauricio Girard, Marianne Lehmann, Qi Yan, Ake T. Lu, Amin Haghani, Juozas Gordevicius, Martin Abba, Rodolfo G. Goya

**Affiliations:** 1 Altos Labs, San Diego, USA; 2 Human Genetics, David Geffen School of Medicine, University of California, Los Angeles, Los Angeles, CA USA; 3 Epigenetic Clock Development Foundation, Torrance, CA USA; 4 CINIBA, School of Medicine, UNLP, La Plata, Argentina; 5 Institute for Biochemical Research (INIBIOLP) - Histology B & Pathology B, School of Medicine, National University of La Plata (UNLP), La Plata, Argentina; 6 Image Analysis Lab (LAI), School of Veterinary Sciences, National University of La Plata, La Plata, Argentina; 7 Vitality in Aging Research Group (VIA), Fort Lauderdale, FL USA

**Keywords:** Hippocampal aging, Spatial memory, OSKM gene therapy, Rejuvenation, OSKM-induced demethylation, Epigenetic age

## Abstract

Several studies have indicated that interrupted epigenetic reprogramming using Yamanaka transcription factors (OSKM) can rejuvenate cells from old laboratory animals and humans. However, the potential of OSKM-induced rejuvenation in brain tissue has been less explored. Here, we aimed to restore cognitive performance in 25.3-month-old female Sprague–Dawley rats using OSKM gene therapy for 39 days. Their progress was then compared with the cognitive performance of untreated 3.5-month-old rats as well as old control rats treated with a placebo adenovector. The Barnes maze test, used to assess cognitive performance, demonstrated enhanced cognitive abilities in old rats treated with OSKM compared to old control animals. In the treated old rats, there was a noticeable trend towards improved spatial memory relative to the old controls. Further, OSKM gene expression did not lead to any pathological alterations within the 39 days. Analysis of DNA methylation following OSKM treatment yielded three insights. First, epigenetic clocks for rats suggested a marginally significant epigenetic rejuvenation. Second, chromatin state analysis revealed that OSKM treatment rejuvenated the methylome of the hippocampus. Third, an epigenome-wide association analysis indicated that OSKM expression in the hippocampus of old rats partially reversed the age-related increase in methylation. In summary, the administration of Yamanaka genes via viral vectors rejuvenates the functional capabilities and the epigenetic landscape of the rat hippocampus.

**Supplementary Information:**

The online version contains supplementary material available at 10.1007/s11357-024-01269-y.

## Introduction

Aging is associated with a progressive increase in the incidence of neurodegenerative diseases, both in laboratory animals and humans. In rats, aging is accompanied by degenerative and/or atrophic processes in the cholinergic system of the forebrain, as well as morphological changes which parallel a reduction in spatial learning capacity [1]. Likewise, there is solid evidence that, in this species, the decline in learning capacity and spatial reference memory that occurs with age is preceded by a multiplicity of structural, cellular, and molecular alterations at the hippocampal level [2, 3], many of which are comparable to those that occur during the aging of the human brain. At the molecular level, gene expression studies in aging rodents have documented significant changes in hippocampal genes related to cholesterol synthesis, inflammation, transcription factors, neurogenesis, and synaptic plasticity [4–8]. In the hippocampus of female rats, 210 genes have been reported to be differentially expressed in aged individuals compared to their young counterparts, with the majority being downregulated [9].

Yamanaka genes, along with other pluripotency genes, possess high therapeutic potential for treating the aged central nervous system affected by various neurodegenerative diseases (for a review see [10]). Recent results revealed that the Yamanaka genes display a dual behavior when expressed continuously in vivo, being regenerative when delivered via viral vectors but highly toxic when expressed in transgenic mice. Thus, it has been reported that delivery of the OSK genes by intravitreally injecting a regulatable adeno-associated viral vector type 2 (AAV2) expressing the polycistron OSK can reverse vision deficits in an experimental model of glaucoma in mice as well as in 11 months old mice showing age-related vison impairment. Fifteen months of continuous expression of the OSK genes in retinal ganglion cells (RGCs) induced neither pathological changes nor proliferation of RGCs. Young- and middle-aged mice injected intravenously with OSK-AAV2 for 15 months did not exhibit any adverse side effects. In contrast, DOX-induced expression of OSK genes in mice transgenic for OSK resulted in rapid weight loss and death, likely due to severe dysplasia in the digestive system [11].

Administering an adenovector to the hypothalamus of young female rats, which carries both the OSKM transcription factors and the green fluorescent protein (GFP) marker, has not only significantly decelerated the pace of reproductive aging but also tripled the fertility rates in 9-month-old females compared to those receiving a placebo vector [12]. Notably, at 9 months of age, female rats are approaching the age of ovulatory cessation, which typically occurs at around 10 months [13]. Inspired by the pioneering results achieved by David Sinclair’s team employing OSK gene therapy in the retina of mice [11], we decided to conduct a medium-term 39-day OSKM gene therapy trial in another brain region: the hippocampus of aged rats. The main goal was to restore learning and spatial memory performance in this animal model. For comparison, we used control groups of similarly aged rats injected with a placebo adenovector.

## Results

### Hippocampal OSKM gene therapy affects spatial memory performance

We used a Tet-Off regulatable HD-recombinant adenoviral vector (RAd) harboring the GFP and Yamanaka genes (Methods, Supplementary Fig. S1) [14].

On day 1, we assigned the animals to three distinct experimental groups and administered the respective adenoviral vectors into the hippocampus. Starting on experimental day 31, we commenced the evaluation of learning and spatial memory performance. Thus, 30 days after the OSKM or control vector injection in the old rats, the three groups were submitted to the Barnes test in order to assess learning performance and spatial memory.

**Fig. 1:**
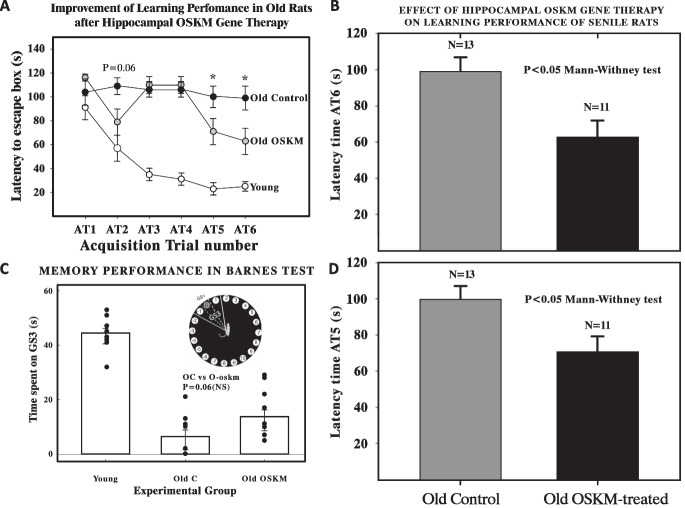
Effect of OSKM genes on learning performance and spatial memory in old rats. **A** The Barnes memory test begins with Acquisition Training, comprising six sessions (acquisition trials or AT). In each AT, a rat is placed at the Barnes platform's center and has 120 s to locate an escape box hidden under one of 20 peripheral holes. **B**,** D** Bar plots show the mean latency time and two standard errors (*y*-axis) in old controls and old treated rats at AT5 and AT6. Old-treated rats show a significant reduction (Mann-Whitney test two-sided *P* < 0.05) in latency time at AT5 (panel** B**) and AT6 (panel** D**) as compared with old controls. Individual data points are reported in Supplementary Table S1. **C** Spatial memory performance in the Barnes test, focusing on the duration rats spend exploring holes − 1, 0, and + 1 in the GS3 sector when the escape box is absent from hole 0. There is a marked fall in spatial memory between young (*N* = 11) and old rats (*N* = 9) but the OSKM treated old rats (*N* = 11) show only a trend (two-sided *P* = 0.06, one-sided *P* = 0.03) towards an improvement as compared with old control animals. The inset shows a diagram of the Barnes platform delineating the GS3 sector

The old controls were always transduced with the GFP vector. Next, we evaluated learning performance and spatial memory (Fig. 1). The Barnes memory test, conducted over six acquisition training sessions (AT), challenges rats to find an escape box within 120 s, concealed under one of 20 holes at the platform’s periphery. Naive at first (AT1), rats learn the box’s location, with young ones typically mastering it by AT3 and improving until a latency time plateau is established. In contrast, older rats demonstrate little progress, showing consistent latency times from AT1 through AT6. An abbreviated protocol based on 3 days of acquisition trials (AT) was used, with two AT conducted per day, totaling six AT. Each AT involved placing a rat in the starting chamber for 30 s, after which the chamber was raised, and aversive stimuli (bright light and high-pitched noise) were activated. The rat was then allowed to freely explore the maze for 120 s. Behavioral performance was recorded using a computer-linked video camera mounted 110 cm above the platform.

In the old rats, 39 days post-OSKM gene therapy in the hippocampus induced a significant improvement in learning performance in the last two acquisition trials (AT5 and AT6, Student’s *t*-test, two-sided *p* < 0.05, Fig. 1B, D) when compared to untreated elderly controls.

Spatial memory in the Barnes test is assessed by tracking the time rats explore adjacent holes (− 1, 0, and + 1) in the GS3 sector without the escape box at hole 0. The time spent in this sector correlates with memory retention; the longer the exploration, the better the spatial memory is presumed to be. A discernible decline in spatial memory is seen between young (*N* = 11) and older rats (*N* = 9), yet OSKM-treated older rats (*N* = 11) indicated a potential improvement against their untreated counterparts, although not conclusively (Student’s *t*-test, two-sided *P* = 0.06, one-sided *P* = 0.03, Fig. 1C). While Fig. 1C showcases spatial *memory* performance, Fig. 1A, B, D illustrates the overall *learning* performance, focusing on the reduction in latency times at AT5 and AT6, indicative of the learning response. Specifically, we were keen on the statistical analysis at these late points, comparing the old controls to the OSKM-treated groups, revealing significant learning enhancements due to the treatment.

### Long-term OSKM gene expression in hippocampal tissue

**Fig. 2:**
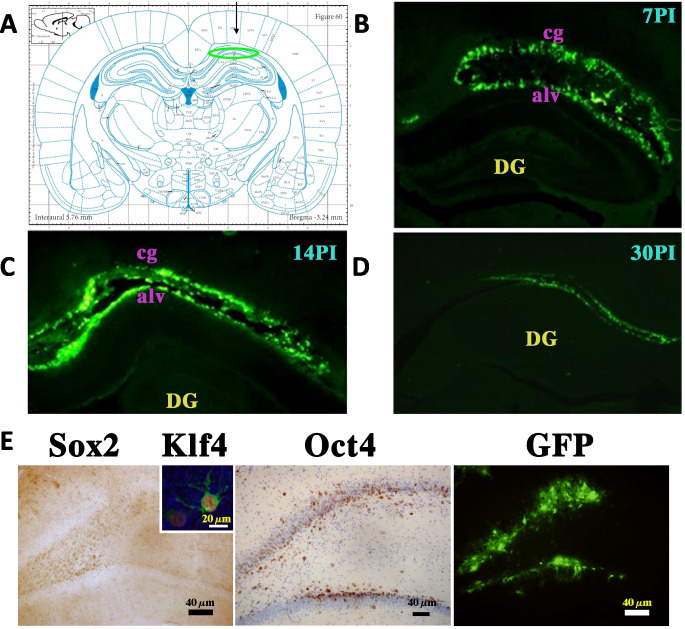
OSKM-GFP expression in the rat hippocampus. **A** The arrow indicates the region of the dentate gyrus in the hippocampus where the OSKM-GFP adenovector was administered. The figure corresponds to Fig. 60 from the Paxinos atlas [33]. In each panel, the GFP signal is consistently localized to the dentate gyrus region of the hippocampus. **B**, **C**, **D** display GFP fluorescence at various time points following vector injection: **B** 7 days post-injection (PI-7), **C** 14 days post-injection (PI-14), and **D** 30 days post-injection (PI-30). Objective: 4× magnification. Scale bars are the same across all panels and are displayed only in the three panels labeled **E**. Four control animals were used in this study. Abbreviations, alv, alveous of the hippocampus; cg, cingulum. **E** Expression of Sox2, KLF4, and GFP in the dentate gyrus of OSKM-GFP adenovector-injected rats. The three panels, from left to right, show expression of Sox2, Oct4, and GFP in the dentate gyrus (DG), 21 days after adenovector injection. The inset shows a hippocampal cell expressing Klf4 and GFP fluorescence. Five treated rats were assessed. Control rats injected with the RAd-GFP placebo vector showed, as expected, the same distribution of GFP expression in the hippocampus. Scale bars indicate the corresponding magnification

Our initial experiments confirmed sustained GFP expression for at least 30 days following the injection of the OSKM adenovector (Fig. 2A–D). Yamanaka gene expression was detectable in the dentate gyrus (DG) for at least 4 weeks post-injection (Fig. 2E).

**Fig. 3:**
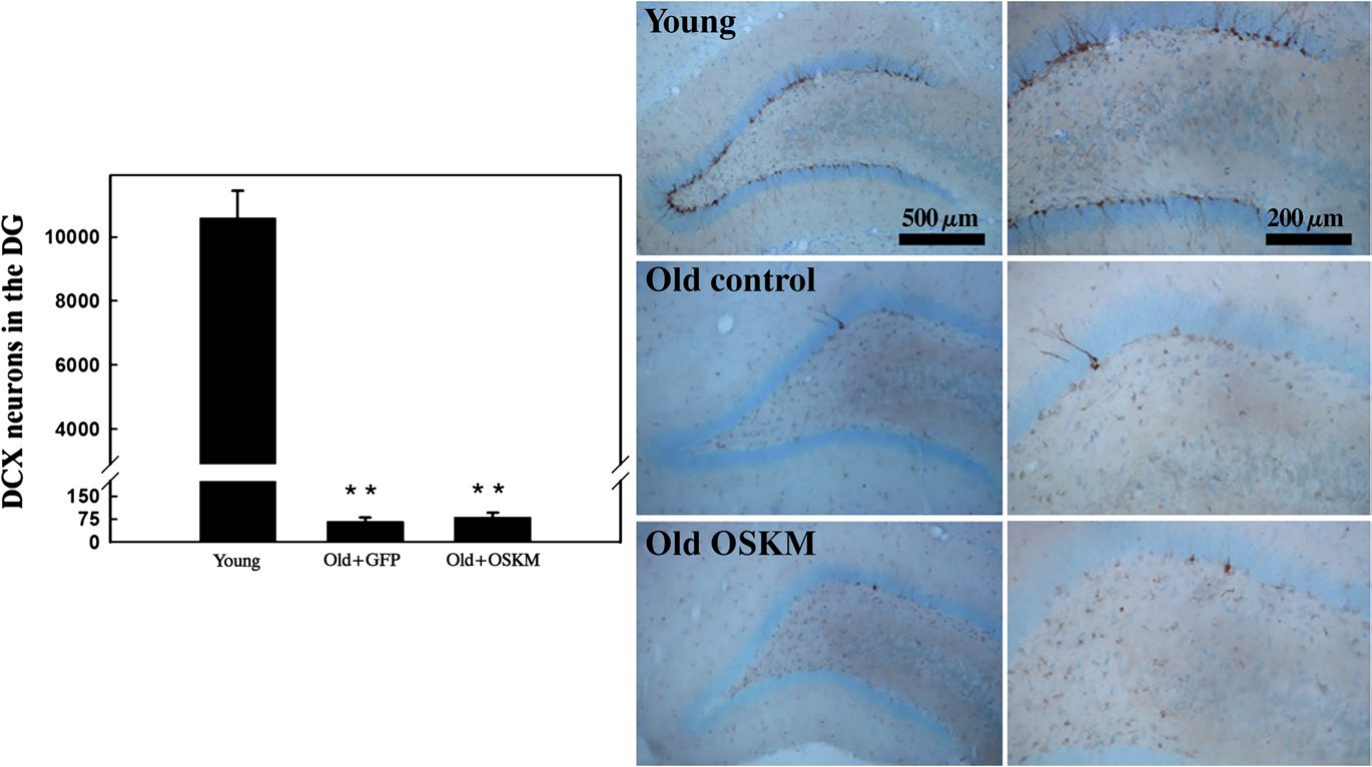
Impact of aging and OSKM gene therapy on the dentate gyrus (DG) granule cell layer. The left bar plot quantifies doublecortin (DCX) positive neurons in the DG of young (*N* = 6 rats), old control (*N* = 5 rats), and old OSKM-treated (*N* = 5 rats) groups. “Old control” refers to aged rats administered with the GFP vector as a control. To the right, we exhibit the comparative decline of DCX neuron counts in aged rats, underscoring the significant decrease attributed to aging. Additionally, no substantial change in the number of DCX neurons was observed in the hippocampus of the old rats following 39 days of OSKM vector treatment. The right-side panels also confirm the absence of pathological changes in the hippocampal tissue of the old rats treated with OSKM for 39 days. Neurons expressing doublecortin (DCX) are identified by their brown labeling. The blue background results from counterstaining with cresyl violet

The granule cell layer of the DG to be particularly intriguing since this layer is home to migratory neurons characterized by the doublecortin (DCX) protein. These neurons, which are also present in the neurogenic subventricular zone (SVZ) of the lateral ventricle, play a crucial role in certain types of spatial memory. With the progression of age, their numbers notably decrease, a trend depicted in Fig. 3 (left panel bar plot). Yamanaka gene therapy did not alter the DCX neuron count in the DG of older rats. As indicated in Fig. 3 (right panel), 39 days of continuous OSKM expression induced no pathological alterations in the hippocampal parenchyma or other brain regions.

Morphological changes were evaluated through visual inspection, while alterations in cell density across various cell types were quantified using a feature of the Image Pro Plus (IPP) v 6.3, software (Media Cybernetics, USA). This function identifies the average size and shape of specific cell types and measures the optical density (OD) of the targeted cell population. Within the hippocampus, the morphology and density of cell populations such as astrocytes (GFAP-labeled) and mature neurons (NeuN-positive cells) remained unchanged in treated aged rats, as compared to their untreated counterparts (Supplementary Fig. S2). Moreover, OSKM treatment did not influence the count of synaptophysin-positive presynaptic boutons in the dentate gyrus (DG), as can be seen from the bottom panel of Supplementary Fig. S2.

### Epigenetic clock analysis of OSKM gene therapy

**Fig. 4:**
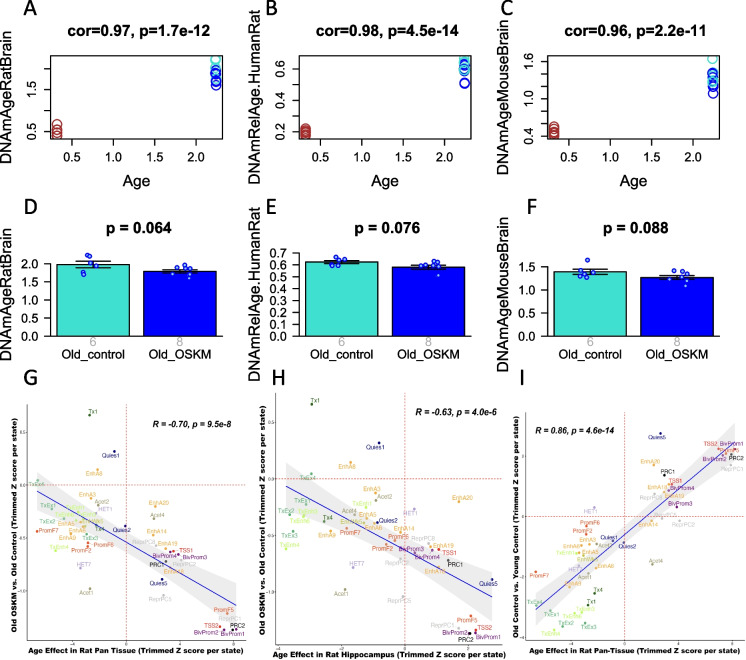
Epigenetic clock analysis of OSKM gene therapy in the rat hippocampus. The columns correspond to three epigenetic clocks: **A, D** Brain clock for rats (units of years). **B**, **E** Human–rat dual species pan tissue clock which estimates relative age (defined as ratio of age divided by maximum species lifespan). **C**,** F** Brain clock for mice estimates chronological age (units of years). **A**,** B**, and **C** present scatter plots with data points color-coded according to the sample condition: brown represents young samples (*N* = 6), blue old samples treated with OSKM (*N* = 8), and turquoise old control samples (*N* = 6). The old controls were always infected with the GFP vector. The bar plots in panels **D**,** E**,** F** depict the average DNAm age estimate with error bars representing one standard error from the mean. Gray numerals atop each bar denote the count of DNA samples for each group. The heading of each panel displays the calculated two-sided Student’s *t*-test *p*-value. The rat clocks are described in [15]. The mouse clocks for brain samples is described [16].** G**, **H** Chromatin state analysis of OSKM rejuvenation (*y*-axis) versus aging effects (*x*-axis). Each point depicted here represents a *Z*-score, derived from the trimmed mean methylation levels of a specific chromatin state (as displayed on the *y*-axis). The chromatin states are defined in accordance with [19]. **G** The axis significance of aging effects in all rat tissues. **H** The *x*-axis significance for age effects throughout all rat tissues, as per the Mammalian Methylation Consortium’s meta-analysis correlation test *Z*-statistic across various rat tissues. **I** Aging effects on chromatin states in all rat tissues (*x*-axis) versus aging effects in the rat hippocampus (*y*-axis)

Epigenetic clocks are statistical models that predict age based on DNA methylation levels at cytosine sites. In this study, we assessed three epigenetic clocks previously developed using independent datasets [15, 16]. The first clock measures chronological age in rat brain samples (Fig. 4A, D). The second clock represents a third-generation model that is applicable to both rats and humans (Fig. 4B, E). Specifically, this interspecies clock calculates relative age, defined as the ratio of chronological age to the maximum lifespan of the species (122 years for humans and 3.8 years for rats). The third clock, initially established for mouse brain samples, is also relevant to rat brains (Fig. 4C). The Pearson correlation coefficient between actual age and DNA methylation age estimates was greater than 0.96 for all three clocks (Fig. 4A, B, and C), indicating their efficacy in differentiating between age groups.

To determine whether OSKM treatment had a rejuvenating effect on the epigenetic age of the rat hippocampus, we confined our analysis to hippocampal samples from older rats, comparing six control samples to eight OSKM-treated samples. The results from all three clocks yielded two-sided Student’s *t*-test *p*-values that were suggestive of rejuvenation (*p* = 0.064 for the rat brain clock, *p* = 0.076 for the dual-species clock, *p* = 0 0.088 for the mouse brain clock, Fig. 4D, E, F). Thus, the results were not significant when analyzed with two-tailed *p*-values, yet they reached significance with one-tailed *p*-values (one-sided *p* < 0.05 for all three clocks). Considering that our initial hypothesis was one-sided, based on prior research demonstrating that OSKM can reverse epigenetic aging, the use of one-tailed *p*-values is arguably more appropriate.

To obtain a more nuanced understanding of the epigenetic landscape associated with treatment-induced methylation changes, we employed a comprehensive universal chromatin state annotation. This annotation was also utilized by our Mammalian Methylation Consortium [17, 18]. This resource, recently made available, was developed from 1032 experiments that identified 32 chromatin marks across more than 100 human cell and tissue types [19]. For each chromatin state, we calculated the trimmed mean methylation levels of all CpGs within that state (see Methods). Analyzing mean methylation levels across chromatin states, rather than individual CpGs, offers several advantages. First, it significantly reduces data volume, shifting the focus from CpGs mapped to the rat genome to 100 chromatin states. Second, it enhances biological interpretability with respect to promoter regions, heterochromatin, and bivalent regulators, among others. Third, this chromatin state analysis approach is conserved across different cell types and species, as shown in previous studies [18, 19].

We found that OSKM treatment reversed the age-associated methylation increase in bivalent regulatory regions and transcription start sites (denoted as BivProm or TSS in Fig. 4G). Conversely, OSKM treatment mitigated the loss of methylation with age in chromatin states associated with heterochromatin (labeled as HET and Quies in Fig. 4G). A notable negative correlation between the aging effects in rat tissues (*x*-axis) and the OSKM rejuvenation effects (indicated by a Pearson correlation coefficient *r* =  − 0.70 in Fig. 4G) indicates rejuvenation at the chromatin state level. In summary, our chromatin state analysis provides strong evidence of epigenetic rejuvenation.

### EWAS

**Fig. 5:**
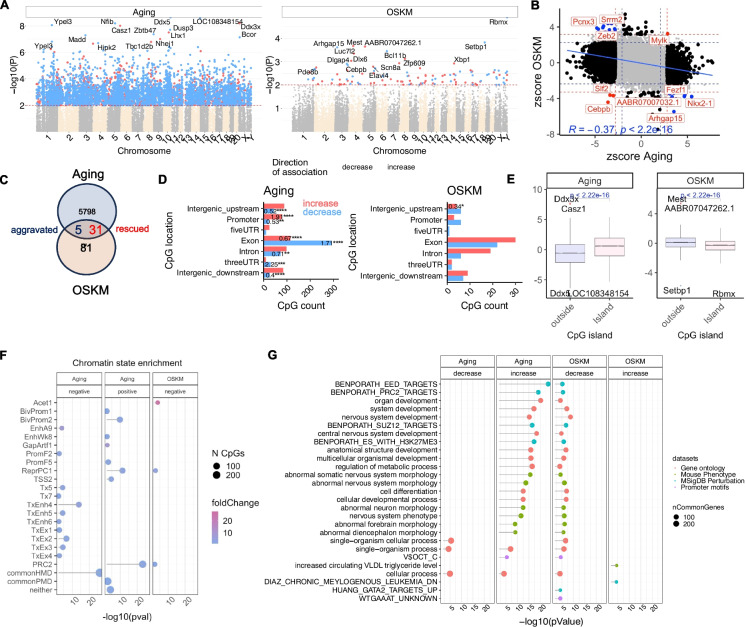
EWAS analysis of OSKM effects vs. age-related methylation in rat hippocampus. **A** Manhattan plots showing EWAS results for chronological age (left) and OSKM treatment effects (right). Genome coordinates are based on the Rattus norvegicus (Rnor_6.0.101). CpGs with *P* < 0.01 are highlighted: red (increased methylation) and blue (decreased methylation). Top 15 CpGs are labeled with adjacent genes. **B** Scatter plots of *Z*-scores for EWAS of age (*x*-axis) vs. OSKM treatment (*y*-axis). Null hypothesis *Z*-statistic follows a standard normal distribution. Red and blue lines: nominal two-sided *p*-values (0.01 and 0.05). Red dots: CpGs whose methylation levels were aggravated/aged by OSKM; blue dots: CpGs rescued/rejuvenated. Top CpGs labeled with adjacent genes. **C** Venn diagram showing overlap of age-associated CpGs with OSKM effects in the hippocampus. CpGs either rejuvenated or aged by OSKM are in the intersection. **D** Location of top CpGs relative to transcriptional start sites, with odds ratios and Fisher exact *p*-values (^*^p < 0.05, ^**^p < 0.01, ^***^p < 0.001, ^****^p < 0.0001). **E** Box plots of EWAS *Z*-statistics vs. CpG island status. Box plots show 25th and 75th percentiles, median line, and 90% whiskers. Student’s *t*-test *p*-values reported. **F** Enrichment of chromatin states for EWAS hits based on StackHMM states in humans [48]. Nominal two-sided *p*-values from hypergeometric tests. PRC2 state based on binding of polycomb repressor complex 2 components (EED, SUZ12, EZH2). **G** Gene set enrichment analysis of top 500 EWAS hits (with *p* < 0.05 per CpG) per methylation direction. Analysis done using GREAT [49] with human Hg19 background and CpGs aligned to the rat genome. Enrichment for gene ontology, mouse phenotypes, promoter motifs, and MsigDB Perturbation terms with nominal *p* < 10^−4^ and minimum overlap of three genes

To study aging effects and OSKM treatment effects at the level of individual CpGs, we conducted epigenome-wide association studies (EWAS, “Methods”). The EWAS *Z*-statistic for age negatively correlated with the treatment effect (Pearson correlation *r* =  − 0.37, *p* < 2.2E-16, Fig. 5B, C), indicating a reversal of age-related methylation changes at the CpG level.

Our EWAS analysis had limited statistical power due to a small sample size (*n* = 8 old OSKM-treated, *n* = 6 old control, and *n* = 6 young samples). To address this, we applied relatively lenient significance thresholds of 0.01 for both treatment and aging effects. We identified 5834 CpGs that differed significantly (nominal, unadjusted *p* < 0.01) between young and old control samples (Fig. 5A). Age-related gains in methylation were significantly enriched in promoter regions, exonic regions (Fig. 5D), and CpG islands (Fig. 5E). Universal chromatin enrichment analysis revealed that age-related methylation gains were observed in bivalent regulatory regions (BivProm2) and target sites of the Polycomb Repressive Complex 2 (Fig. 5F, G), consistent with results from previous pan-mammalian aging studies [18]. Conversely, age-related losses in methylation were significantly enriched in intergenic regions and introns (Fig. 5D).

Focusing on the old hippocampal samples, we evaluated the effects of OSKM treatment. This analysis identified 117 CpGs with significant changes due to the treatment (*p* < 0.01, Fig. 5A). These CpGs were statistically enriched in intergenic regions, with the majority (52 CpGs) located in exons. The most significant OSKM-related CpG was near the Rbmx, Setbp1, and Mest genes (Fig. 5A, E). Interestingly, OSKM-related losses in methylation were significantly enriched in PRC2 target regions (Fig. 5F, G), suggesting a reversal of age-related gains in methylation in these regions. Overall, these EWAS results are consistent with our chromatin state analysis and epigenetic clock studies, suggesting that OSKM treatment restores the youthful methylome.

## Discussion

Previous studies have demonstrated the rejuvenative effects of partial, cyclic in vivo reprogramming using OSKM genes in human cells [20]. Partial reprogramming studies in transgenic mice have been found to extend survival and partially rejuvenating some tissues in progeric mice [21–23]. Cyclic partial reprogramming in middle-aged, OSKM-transgenic mice partly reversed age-dependent reduction in hippocampal histone H3K9 trimethylation, enhancing granular cell migration in the DG and significantly improving performance in object recognition tests without extending their life [24].

With our OSKM-GFP adenovector, we implemented long-term regenerative gene therapy in the hypothalamus of young females with the goal to extend fertility in the treated animals. This hypothalamic OSKM gene therapy in young rats has shown to slow age-related fertility decline [12]. Similarly, transgenic OSKM mice submitted to a single short reprogramming in early life showed protective effects, extending lifespan by 15% and functional capacities [25]. These, heterozygous progeric mice submitted post-puberty to moderate OSKM gene expression for 2.5 weeks exhibited body and functional improvements throughout life [25].

A preprint has documented that 124-week-old mice, upon receiving cyclic intravenous treatments with an AAV9-based two-vector gene therapy system targeting OSK genes, exhibited an average lifespan increase of 2 months [26]. This suggests that Yamanaka gene therapy can prolong life and is linked to slower epigenetic aging. In 12-month-old OSKM transgenic mice, such therapy boosted regenerative capabilities of the pancreas and muscle, triggering cell proliferation critical for tissue homeostasis, countering age-related decline [21].

While these findings align with our study, our approach leverages the continuous, long-term expression enabled by Yamanaka gene therapy, which does not induce adverse effects on cells or tissues and promises to exhibit stronger, more prolonged regenerative effects. Contrary to the rapid onset of adverse effects such as visceral teratomas that can occur within a week due to the expression of Yamanaka factors in transgenic mice, our observations did not show any negative outcomes for a period of 39 days, which is a substantially extended timeframe. Nevertheless, the possibility of long-term adverse effects remains undetermined.

In our study, we specifically investigated the effects of OSKM gene delivery on hippocampal DNA methylation. We found suggestive evidence of epigenetic rejuvenation, corroborated by two separate epigenetic clocks designed specifically for rat and mouse brain samples. These findings align with numerous studies that have also reported signs of epigenetic rejuvenation in human and mouse cells and tissues following OSKM or OSK application [11, 20–22, 27, 28]. While our sample size was limited (*n* = 8 old OSKM-treated samples versus *n* = 6 old controls), these epigenetic clocks, trained using independent data, provide insights due to their accuracy.

Our epigenetic clock studies provide preliminary evidence of epigenetic rejuvenation, a concept further supported by the chromatin state analysis. These detailed investigations suggest that OSKM treatment can counteract age-related methylation changes in specific chromatin states, further substantiating the idea of epigenetic rejuvenation.

The impact of OSKM (or OSK) gene expression on the DNA methylation landscape in the hippocampus of aged animals (or humans) remains largely unexplored. Our prior research has demonstrated that older rats exhibit increased methylation in hippocampal DNA compared to their younger counterparts [29]. Furthermore, pathway enrichment analysis has shown that genes involved in neuron fate commitment, brain development, and central nervous system development are notably enriched in hypermethylated CpGs in these older animals [29]. The present study provides further insights into the impact of OSKM gene therapy on the DNA methylation landscape in the hippocampus of old animals. In effect, our findings suggest that while in hippocampal DNA, aging prevalently hypermethylates some genes, 56% of the genes hypomethylated by OSKM gene therapy in old hippocampi overlap with the genes hypermethylated during aging, Consequently, the hippocampal genes hypermethylated during aging and hypomethylated by OSKM in old hippocampal DNA, inversely affect the same processes, namely, nervous system development, neuron generation, and differentiation. This observation suggests that in the old hippocampus, the OSKM genes are driving the epigenome backwards along the same molecular path followed during aging.

In the context of the slight epigenetic rejuvenation observed in the treated old hippocampi, it is worth noting that only a small proportion of hippocampal cells would have been directly rejuvenated by our OSKM-GFP vector. Additionally, those few cells expressing the OSKM genes might have created a rejuvenating environment in the hippocampal tissue, contributing to a mild reduction in the epigenetic age of the whole region.

Our findings confirm previous results [11] that viral vector-mediated delivery of the Yamanaka genes in the brain has strong regenerative effects in rodents without adverse side effects. Our HD OSKM-GFP adenovector offers an effective gene delivery tool for the long-term expression of large polycistronic gene constructs. As mentioned above, we have recently demonstrated that this same adenovector can attenuate the age-related decline in fertility when expressed in the hypothalamus of young female rats for 5.8 months [12].

Given the inherent rejuvenating properties of the Yamanaka genes [20–22, 28, 30, 31], studies examining their functional effects in adult and old animals present a promising research avenue in regenerative medicine.

## Conclusions

In vivo partial reprogramming is known to improve age-associated molecular changes during physiological aging in mice [22]. But OSKM transgenic mice undergoing cyclic partial reprogramming face two significant challenges: the in vivo toxicity of Yamanaka genes and the observation that primarily progeric mice show substantial functional improvements with OSKM treatment, while normal OSKM transgenic mice show less pronounced benefits [24].

The use of OSK or OSKM gene therapy, as pioneered by David Sinclair’s laboratory, has emerged as a more effective approach in wild-type rodents [11]. Our research in the hippocampus of aged rats corroborates the advantages of OSKM gene therapy. To date, notable applications of OSKM or OSK gene therapy in vivo include a study on retinal aging in a non-transgenic mouse model [11], enhanced fertility in wild-type female rats via hypothalamic OSKM gene therapy [12], lifespan extension in aged wild-type mice through intravenous OSK gene therapy [26], and the results of our current study. Overall, our findings add to the growing evidence supporting the potential of in vivo Yamanaka gene therapy as a rejuvenation strategy.

## Methods

### Material and methods

#### Adenoviral vectors

We used a Tet-Off regulatable HD-recombinant adenoviral vector (RAd) harboring the GFP and Yamanaka genes using a commercial kit (Microbix Inc., Ontario, Canada) that provides the shuttle plasmid pC4HSU, the helper adenovirus H14 and the HEK293 Cre4 cell line. A full description of the adenovector can be found in [14]. Briefly, we cloned a construct harboring the bicistronic tandem Oct4-f2A-Klf4-ires-Sox2-p2A-cMyc (known as hSTEMCCA, generously provided by Dr. G. Mostoslavsky, Boston University) under the control of the bidirectional Tet-Off promoter PminCMV-TRE-PminCMV which on the second end is flanked by the gene for green fluorescent protein (GFP).

The hSTEMCCA cassette harbors the four Yamanaka genes grouped in pairs placed downstream and upstream of an internal ribosome entry site (IRES). In turn, each pair of genes is separated by a type 2A CHYSEL (cis-acting hydrolase element) self-processing short sequence which causes the ribosome to skip the Gly-Pro bond at the C-terminal end of the 2A sequence, thus releasing the peptide upstream the 2A element but continuing with the translation of the downstream mRNA sequence. This allows near stoichiometric co-expression of the two cistrons flanking a 2A-type sequence [32]. The whole expression cassette (STEMCCA cassette 10,065 bp) was cloned into the pC4HSU HD shuttle, giving rise to pC4HSU-STEMCCA-tTA. The pC4HSU HD shuttle consists of the inverted terminal repeats (ITRs) for Ad 5 virus, the packaging signal and part of the E4 adenoviral region plus a stuffer non-coding DNA of human origin which keeps a suitable size (28–31 Kbp) of the viral DNA.

The linearized DNA backbone of the new HD-RAd **(**Supplementary Fig. S1**)** was transfected in Cre 293 cells. For expansion, the helper H14 adenovirus was added to the cell cultures at a multiplicity of infection (MOI) of 5. In H14, the packaging signal is flanked by lox P sites recognized by the Cre recombinase expressed by the 293 Cre4 cells. Therefore, the helper virus provides in trans all of the viral products necessary for generation of the desired HD-RAd. The infected 293 Cre4 cells were kept until cytopathic effect (CPE) was evident. Cells were lysed and clear lysates mixed with H14 helper virus and added to a fresh culture of Cre4 293 cells at MOI 1. When CPE appeared, passage 2 (P2) cell lysates were prepared. This iterative co-infection process was carried on five more times in order to generate enough HD-RAd particles for virus purification. The newly generated HD-RAd was purified by ultracentrifugation in CsCl gradients. We used a control RAd harboring the hGFP gene.

### Animals and in vivo procedures

Female Sprague–Dawley rats initially aged 3.5 months (young) and 25.3 (old) months were used. The animals were raised in our institution (INIBIOLP) and housed in a temperature-controlled room (22 ± 2 °C) on a 12:12-h-light/dark cycle (lights on from 7 to 19 h). Food and water were available ad libitum. All experiments with animals were performed according to the Animal Welfare Guidelines of NIH (INIBIOLP’s Animal Welfare Assurance No A5647-01).

### Experimental design for long-term OSKM gene therapy in the hippocampus

We initiated the study by distributing rats into three groups: young intact (*N* = 12), old control (*N* = 16) injected with RAd (GFP), and old OSKM (*N* = 17) injected with RAd OSKM-GFP vector. All old controls received the GFP vector. Control and treated female rats received a single injection of vectors into both the left and right hippocampi. Cognitive tests commenced 31 days post-injection. Learning and memory performance assessments began on day 31 post-injection. We chose 30 days post-treatment because we previously determined that vector expression in the hippocampus (GFP fluorescence) remains detectable for at least 30 days. Our aim was to allow the OSKM genes to act for as long as possible, assuming that prolonged exposure to the OSKM genes may enhance their regenerative effects on memory. Future studies are needed to delineate the time-course of the regenerative effects of OSKM on memory performance.

Attrition of subjects occurred due to complications from stereotaxic surgery and motor deficits leading to falls during platform exploration, necessitating the elimination of some old animals. The revised cohort consisted of 12 young, 13 old controls, and 11 OSKM-treated old rats for Fig. 1A, B. For spatial memory tests in Fig. 1C, the numbers were adjusted to 11 subjects in both the young and old control groups and 9 in the OSKM-treated old group.

For the DNA methylation analysis, sample exclusion was based on DNA quality control failures (described below). The final analysis was conducted on 20 DNA samples using the mammalian 40 K array: six young, eight old OSKM-treated, and six old controls.

### Brain processing

Animals were placed under deep anesthesia and perfused with phosphate-buffered para-formaldehyde 4% (pH 7.4) fixative. The brains were rapidly removed and stored in para-formaldehyde 4% (pH 7.4) overnight (4 °C). Finally, brains were maintained in cryopreservative solution at − 20 °C until use. For immunohistochemical assessment, brains were cut coronally in 40-µm-thick sections with a vibratome (VT1000S; Leica Microsystems, Wetzlar, Germany).

### Immunohistochemistry

Details are reported in the Supporting Information.

### Immunofluorescence

The immunofluorescence technique was based on the following primary antibodies, anti-synaptophysin antibody [YE269] (ab32127), anti-GFAP monoclonal antibody 1:500 (G3893, Sigma see above) and anti NeuN-Mouse monoclonal antibody [1B7] (ABCAM Cat# ab104224), and mouse anti-KLF4 polyclonal antibody (Invitrogen Cat #PA5-27441).

Once incubated with the primary antibodies, slides were incubated with either goat anti mouse Alexa Fluor™ 647 (Invitrogen Cat # A-21237) or Alexa Fluor 647 AffiniPure F(ab')₂ fragment goat anti-rabbit IgG (H + L) (Jackson Labs, 111–606-144), overnight at 4 °C in a dark chamber. Then, sections were rinsed threefold in PBS and counterstained for 15 min with DAPI. Control negative sections were prepared by omitting the primary antibody.

### Image analysis of DCX cells

In each hippocampal block, one every six serial sections was selected in order to obtain a set of non-contiguous serial sections spanning the dorsal hippocampus (240 µm apart). The number of cells was assessed in the dorsal hippocampus which is located between coordinates − 2.8 to − 4.5 mm from the bregma [33]. Images were captured using an Olympus video camera (DP70. Tokyo, Japan) attached to an Olympus BX-51 microscope, using a 40× magnification objective. The total number of cells was estimated using a modified version of the optical dissector method [34–36]. Individual estimates of the total bilateral neuron number (*N*) for each region were calculated according to the following formula: *N* = RQΣ · 1/ ssf · 1/asf · 1/tsf, where RQΣ is the sum of counted neurons, ssf is the section sampling fraction, asf is the area sampling fraction, and tsf is the thickness sampling fraction.

Immunofluorescence images were captured using a laser scanning confocal microscope (Olympus FV1000, Japan). Neurons, neuropil, and glial cells labeled with Alexa 647 were excited using a 635-nm solid-state laser. All nuclei stained with DAPI were excited using a 405-nm solid-state laser. GFP expression was excited using a 473-nm solid-state laser.

#### Stereotaxic injections

Rats were anesthetized with ketamine hydrochloride (40 mg/kg; ip) plus xylazine (8 mg/kg; im) and placed on a stereotaxic apparatus. In order to access the dentate gyrus (DG) of the dorsal hippocampus, the tip of a 26G needle fitted to a 10-µl syringe was brought to the following coordinates relative to the bregma: AP: 3.5 mm; ML: ± 2 mm; DV: 4 mm [33] and a 3-µl vector suspension per side was slowly injected (1 µl/min).

### Spatial memory assessment

#### Description of the Barnes maze protocol used

The modified Barnes maze protocol used in this study was based on a previously reported procedure [3, 35, 36]. It consists of an elevated (108 cm to the floor) black acrylic circular platform, 122 cm in diameter, containing 20 holes around the periphery. The holes are of uniform diameter (10 cm) and appearance, but only one hole is connected to a black escape box (tunnel). The escape box is 38.7 cm long × 12.1 cm wide × 14.2 cm in depth and it is removable. A white squared starting chamber (an opaque, 20 cm × 30 cm long, and 15 cm high, open-ended chamber) was used to place the rats on the platform. Four proximal visual cues were placed in the room, 50 cm away from the circular platform. The escape hole was numbered as hole 0 for graphical normalized representation purposes, the remaining holes being numbered 1 to 10 clockwise, and − 1 to − 9 counterclockwise **(**Fig. 1C, inset). Hole 0 remains in a fixed position, relative to the cues in order to avoid randomization of the relative position of the escape box. A 90-dB white-noise generator and a white-light 500 W bulb provided the escape stimulus from the platform. We used an abbreviated protocol based on 3 days of acquisition trials (AT), making a total of 6 AT. An AT consists of placing a rat in the starting chamber for 30 s, the chamber is then raised, and the aversive stimuli (bright light and high pitch noise) are switched on and the rat is allowed to freely explore the maze for 120 s.

The behavioral performances were recorded using a computer-linked video camera mounted 110 cm above the platform. The video-recorded performances of the subjects were measured using the Kinovea v0.7.6 (http://www.kinovea.org) and Image Pro Plus v5.1 (Media Cybernetics Inc., Silver Spring, MD, USA) software. The behavioral parameters assessed were as follows.

- Escape box latency: time (in s) spent by an animal since its release from the start chamber until it enters the escape box in an AT.
- Goal sector (GS): the area of the platform corresponding to a given number of holes. GS3, is the area corresponding to holes − 1, 0, + 1.
- Spatial memory assessment: One day after the last AT is done, a trial, known as probe trial (PT), where the escape box has been removed, is performed, its purpose being to assess the permanence time in GS3, calculated taking the time spent by the rats in the area covered by the three holes during the PT.

### Hippocampus dissection

Five rats from each group were sacrificed by decapitation, the brain was removed carefully, severing the optic nerves and pituitary stalk and placed on a cold plate. The hippocampus was dissected from cortex in both hemispheres using forceps. This dissection procedure was also performed on the anterior and posterior blocks, alternatively placing the brain caudal side up and rostral side up. After dissection, each hippocampus was immediately placed in a 1.5-ml tube and momentarily immersed in liquid nitrogen, then stored at − 80 °C for DNA extraction.

### Hippocampal DNA extraction

Hippocampal DNA was extracted using an automated nucleic acid extraction platform called QIA cube HT (Qiagen) with a column-based extraction kit, QIA amp 96 DNA QIA cube HT kit (Qiagen).

### Purification and methylation analysis of genomic DNA

DNA was extracted from 24 rat hippocampi using the Qiagen DNeasy blood and tissue kit, selecting 20 samples with optimal 260/280 ratios near 1.8, indicative of minimal protein contamination, for analysis. The bisulfite conversion of *N* = 20 DNA samples was performed using the EZ methylation kit (Zymo Research, D5002).

### Epigenetic age estimates based on methylation clocks

The converted genomic DNA was analyzed by the mammalian methylation array (HorvathMammalMethylChip40) [37]. The same mammalian methylation array platform and statistical pipeline have been employed to construct epigenetic clocks for numerous mammalian species, as well as for several non-mammalian ones [38–46]. Here, we used three previously published epigenetic clocks: rat brain clock that estimates chronological age and a dual species clock (human rat clock) for estimating relative age. The software code for these rat clocks, which were constructed in independent data, can be found in [15]. Briefly, penalized regression models were created with the R function “glmnet” [47]. We also evaluated a mouse clock for estimating chronological age in mouse brain samples [16].

### Methylation data

The description of EWAS and chromatin state analysis can be found in Supplementary Information.

## Supplementary Information

Below is the link to the electronic supplementary material.

Supplementary file1 (DOCX 4838 KB)

## Data Availability

The methylation data can be found at Gene Expression Omnibus GSE252401. The mammalian array platform is distributed by the non-profit Epigenetic Clock Development Foundation (https://clockfoundation.org/).
