# Supplementary material for: Cognitive rejuvenation in old rats by hippocampal OSKM gene therapy
Source: GeroScience. 2024 Jul 22;47(1):809–23. doi: 10.1007/s11357-024-01269-y (PMC11872836; doi:10.1007/s11357-024-01269-y)
Supplement: Supplementary file 1 — Supplementary file1 (DOCX 4838 KB) [file 11357_2024_1269_MOESM1_ESM.docx]

**Supplementary Information for the article entitled**

**"COGNITIVE REJUVENATION IN OLD RATS BY HIPPOCAMPAL OSKM GENE THERAPY"**

Supplementary Figures and Supplementary Methods.

**
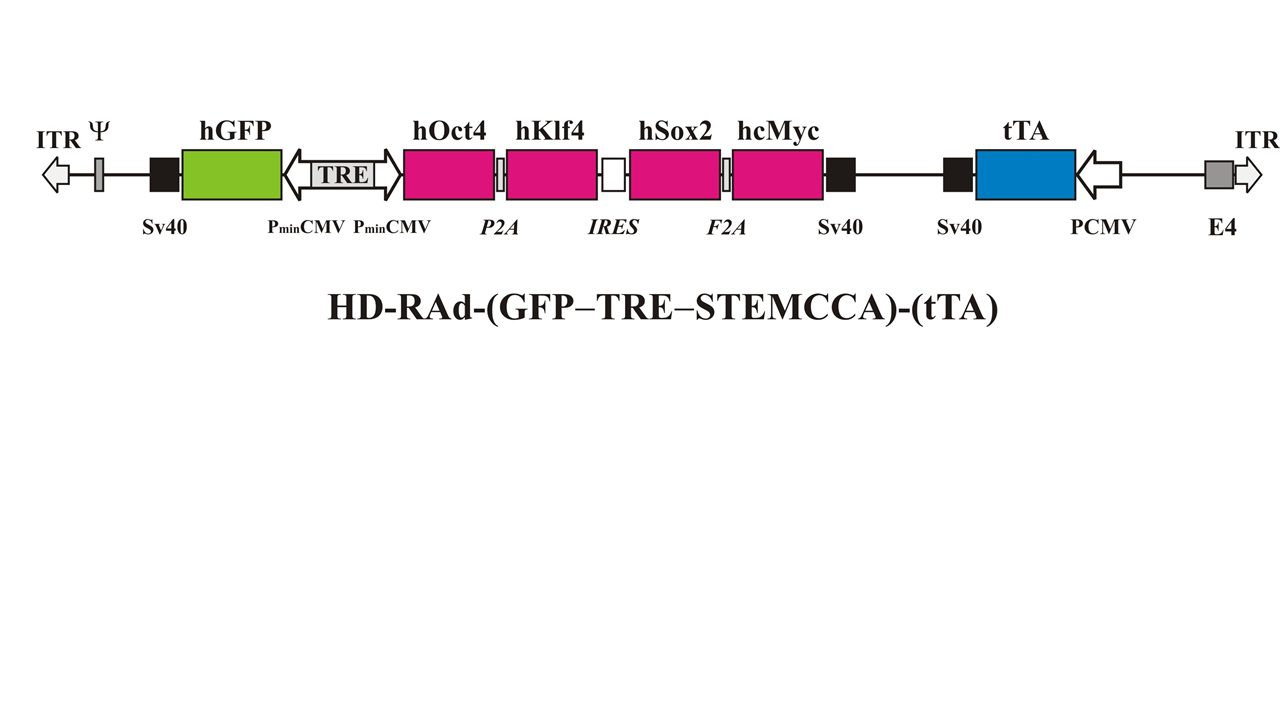
**

**Supplementary Figure S1.** The figure illustrates the basic components of HD-RAd-STEMCCA-GFP-Tet-Off genome.

Abbreviations- GFP: humanized Green Fluorescent Protein; TRE: Tetracycline responsive element; tTA: chimeric regulatory protein; PminCMV: cytomegalovirus minimal promoter; P2A &, F2A, 2A CHYSEL (cis-acting hydrolase element) self-processing short sequences. SV40pA: polyadenylation signal; ITR: inverted terminal repeats; ψ: packaging signal.

**
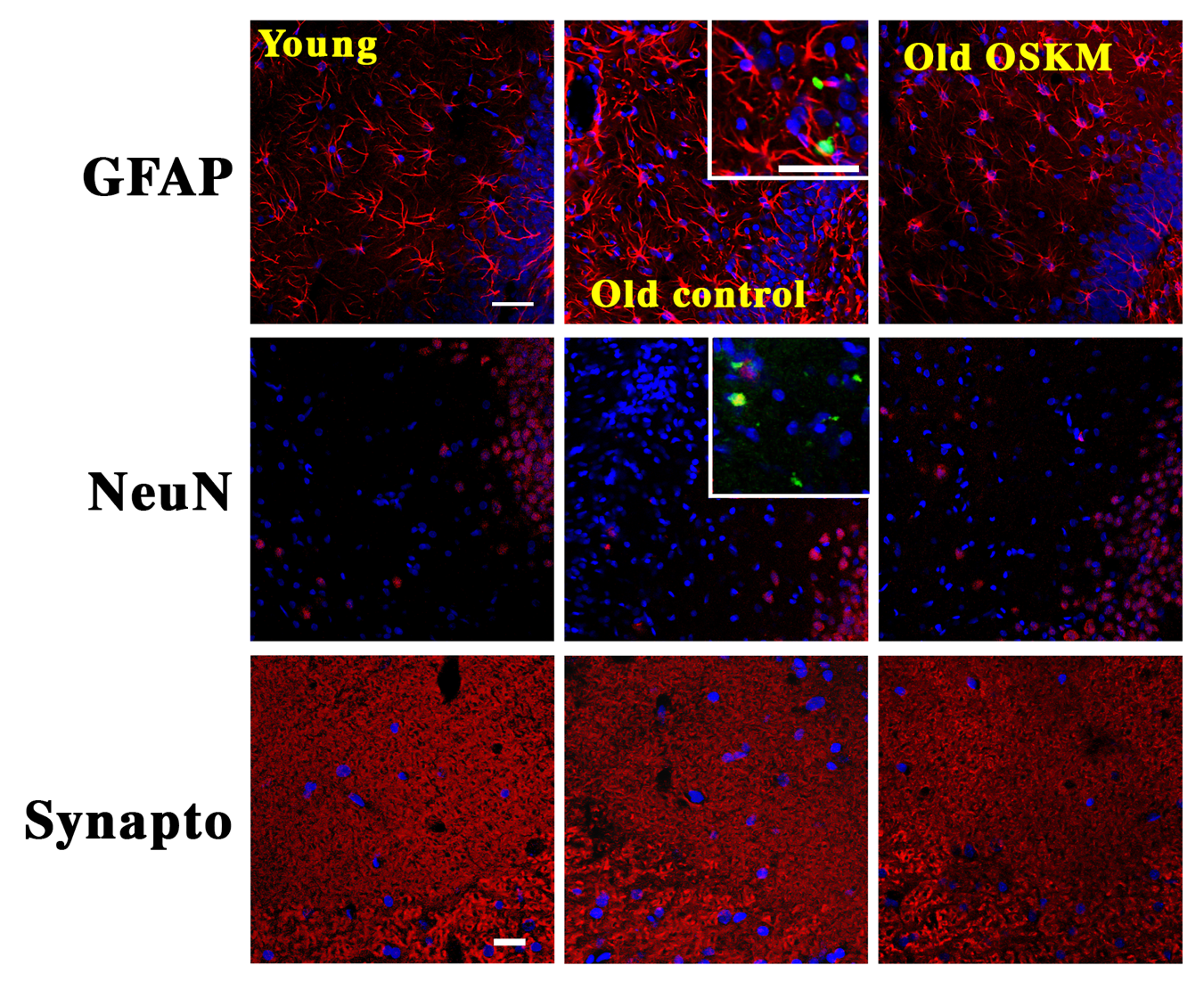
**

**Supplementary Figure S2. Effect of OSKM gene therapy on the hippocampal astrocyte and mature neuron population as well as on synaptic button density in old rats.** After 39 days of OSKM vector injection in the dorsal hippocampus of old rats, there were no detectable changes in the astrocyte and mature neuron populations. Synaptophysin-positive presynaptic bouton density was not affected either. Insets show astrocytes or NeuN neurons (red color) expressing the GFP gene reporter. Seven treated old rats were assessed.

The scale bars for both NeuN and GFAP panels are consistent across the inset and main panels. The scale bars provided in the GFAP panel are applicable to the NeuN panel as well, whereas the Synaptophysin panel uses a different scale bar. The statistical analysis conducted using the IPP software has not been included in the display.


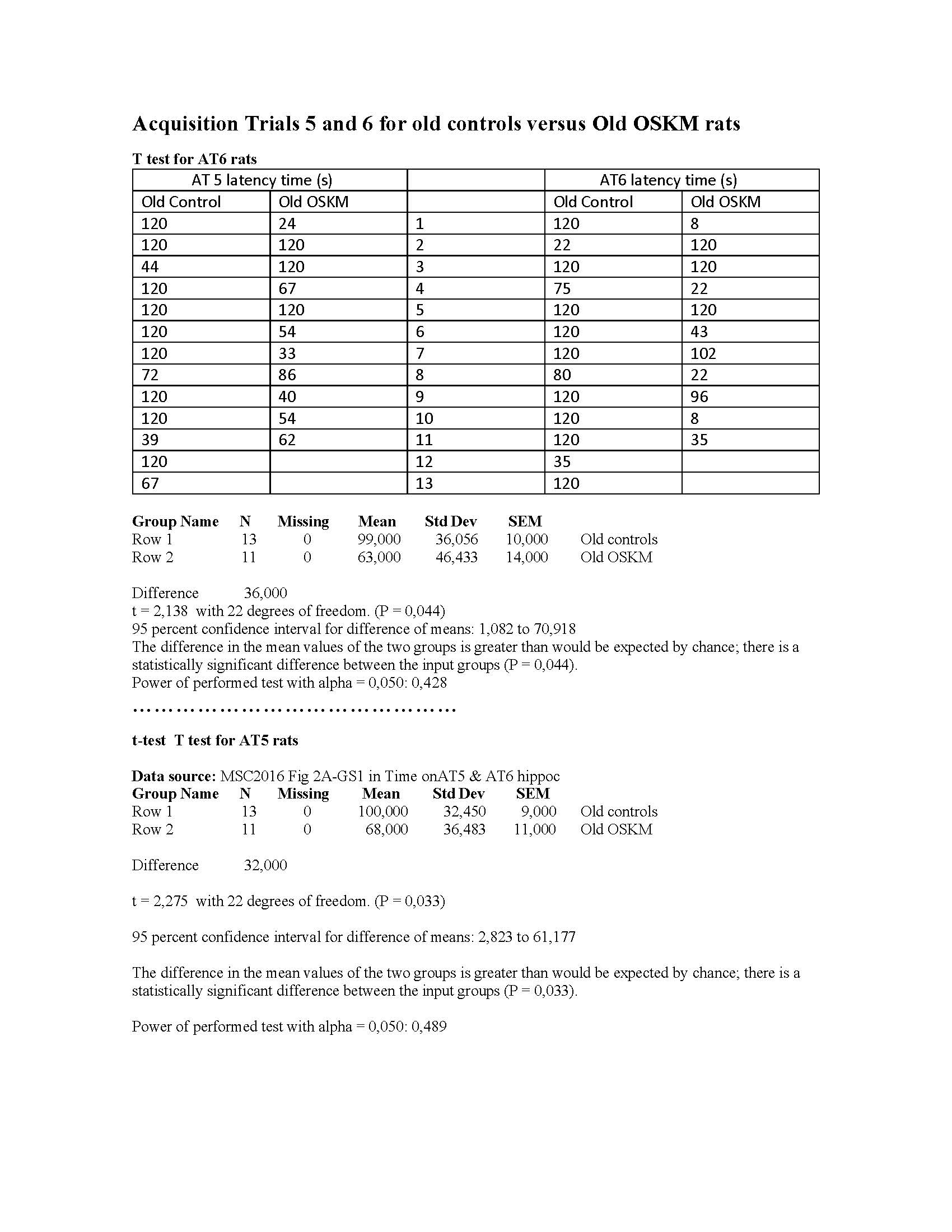


**Supplementary Table S1. Individual data points underlying Figure 1B,D.**

Further, we report the results of the two Student t-tests performed by the software (Sigma Plot 10).

**Supplementary Methods**

**Immunohistochemistry**

All immunohistochemical techniques were performed on free-floating sections. For each animal, separate sets of sections were immunohistochemically processed using anti-glial fibrillary acidic protein (GFAP) monoclonal antibody 1:500 (G3893, Sigma, Saint Louis, Missouri), anti-doublecortin (DCX) goat polyclonal antibody 1:250 (c-18, Santa Cruz Biotech., Dallas, Texas), mouse anti-hOct4 (1:10, BD Pharmingen, San Jose, CA), mouse anti-hSox2 (1:40, BD Pharmingen, San Jose, CA) and mouse anti-hc-Myc (1:50, BD Pharmingen, San Jose, CA). One limitation to note is that the commercial antibodies for the Yamanaka factors resulted in unexpected immunolabeling within the hippocampus. Although the suppliers confirm reactivity with rat and mouse tissues, they do not specify the extent of the antibodies' specificity

For detection, the Vectastain® Universal ABC kit (1:500, PK-6100, Vector Labs., Inc., Burlingame, CA, USA) employing 3, 3-diamino benzidine-tretrahydro-chloride (DAB) as chromogen, was used. Briefly, after overnight incubation at 4ºC with the primary antibody, sections were incubated with biotinylated horse anti-mouse antiserum (1:300, BA-2000,Vector Labs.) or horse anti-goat antiserum (1:300, BA-9500, Vector Labs), as appropriate, for 120 min, rinsed and incubated with avidin-biotin-peroxidase complex (ABC Kit) for 90 min and then incubated with DAB. Sections were counterstained with Nissl method (0.5% cresyl violet solution at 37ºC for 10 minutes) to visualize anatomical landmarks and mounted with Vectamount (Vector Labs) to use for image analysis.

**EWAS**

For our EWAS, we adhered to the same bioinformatics pipeline employed by the Mammalian Methylation Consortium (Haghani et al., 2023; Lu et al., 2023). The corresponding software scripts are available for download from the GitHub page linked to these articles (Haghani et al., 2023; Lu et al., 2023). We maintained this pipeline to ensure compatibility with our previous publications. The EWAS was confined to CpGs on the mammal array 40 that mapped to the rat genome, analyzing each individual CpG site in relation to the trait of interest. Genome coordinates were based on the Rattus norvegicus (Rnor_6.0.101).

Correlation test results for age were obtained using the R function corAndPvalue from the WGCNA package (Langfelder & Horvath, 2008), which provided the Pearson correlation coefficients for each test. Z-scores for the EWAS of age were calculated using the Fisher transformation of p-values. The EWAS for OSKM was conducted using a linear association analysis in the Limma package, with chronological age included as a covariate in the model. Z-scores of the linear model were calculated using the formula

z = (t - mean(t))/SD(t).

Enrichment of chromatin states for EWAS hits was based on Universal StackHMM states in humans (Vu & Ernst, 2022). Nominal two-sided p-values were obtained from hypergeometric tests. The PRC2 state was defined by the binding of Polycomb Repressor Complex 2 transcription factors (EED, SUZ12, EZH2) as detailed in (Lu et al., 2023).

Functional enrichment analysis of the EWAS results was conducted using GREAT (McLean et al., 2010) with a human Hg19 background and CpGs aligned to the rat genome. We used up to 500 of the most significant CpGs per direction as input, restricting the analysis to CpGs with a nominal two-sided p-value of less than 0.05.

**Trimmed mean based on universal chromatin states**

For each chromatin state, which encompasses several hundred CpGs, we calculated a single representative value for each DNA sample by computing the trimmed mean of the CpGs associated with that chromatin state. This process yields a mean value for each rat tissue sample, resulting in a 20-component vector, with each component representing a distinct rat sample. The trimmed mean for each chromatin state was associated with the 'Condition' variable, which is also comprised of 20 components. These components classify the samples into three groups: young controls, old controls, and old OSKM-treated samples.

To annotate our age-related CpGs based on chromatin states, we assigned a state for all our mammalian CpGs based on a recently published universal ChromHMM chromatin state annotation of the human genome (Vu & Ernst, 2022). The underlying hidden Markov model (HMM) was trained with over 1,000 datasets of 32 chromatin marks in more than 100 cell and tissue types. This model then produced a single chromatin state annotation per genomic position that is applicable across cell and tissue types, as opposed to producing an annotation that is specific to one cell or tissue type. A total of 100 distinct states were generated and categorized into 16 major groups according to the parameters of the model and external genome annotations.

To form the trimmed mean DNA methylation per chromatin state, we first filtered CpGs for each state (i.e., “trimmed”). Based on over 600 publicly available rat samples including both brain (e.g., hippocampus, hypothalamus, etc.) and proliferative tissues (e.g., blood, skin, etc.), we calculated the average methylation level of each CpG. We then compared the methylation level of each CpG with the distribution of methylation levels of the chromatin state to which it belongs. A CpG was filtered out if its methylation level exceeded three times the interquartile range (IQR) of the first quartile (Q1 × IQR) or the third quartile (Q3 + 3 × IQR) of the methylation level of the corresponding chromatin state. To further remove CpGs that are not relevant to age, we conducted epigenome-wide association study (EWAS) based on all rat samples and filtered out CpGs that were not correlated with chronological age (raw p-value >= 0.05).

After the trimming step, there are 9,620 CpGs remaining that are shared between the Illumina Mammalian 40K Array and the Mammal 320K Mouse Array. We used these 9620 CpGs to calculate the mean methylation for each chromatin state and to perform chromatin state differential methylation analysis. The Limma package was used to assess differential methylation by fitting linear models to the methylation data for specific group comparisons: Old Control versus Old OSKM hippocampus. We also conducted differential methylation analysis of chronological age based on available rat samples and used the summary statistics as reference to assess whether OSKM treatment could reverse the aging effect.

**REFERENCES**

Haghani, A., Li, C. Z., Robeck, T. R., Zhang, J., Lu, A. T., Ablaeva, J., . . . Almunia, J. (2023). DNA methylation networks underlying mammalian traits. *Science, 381*(6658), eabq5693.

Langfelder, P., & Horvath, S. (2008). WGCNA: an R package for weighted correlation network analysis. *BMC Bioinformatics, 9*(1), 559.

Lu, A. T., Fei, Z., Haghani, A., Robeck, T. R., Zoller, J. A., Li, C. Z., . . . Horvath, S. (2023). Universal DNA methylation age across mammalian tissues. *Nature Aging*. doi:10.1038/s43587-023-00462-6

McLean, C. Y., Bristor, D., Hiller, M., Clarke, S. L., Schaar, B. T., Lowe, C. B., . . . Bejerano, G. (2010). GREAT improves functional interpretation of cis-regulatory regions. *Nature biotechnology, 28*(5), 495-501.

Vu, H., & Ernst, J. (2022). Universal annotation of the human genome through integration of over a thousand epigenomic datasets. *Genome biology, 23*, 1-37.
